# Oral fucoidan improves muscle size and strength in mice

**DOI:** 10.14814/phy2.14730

**Published:** 2021-02-01

**Authors:** Sally E. McBean, Jarrod E. Church, Brett K. Thompson, Caroline J. Taylor, J. H. Fitton, Damien N. Stringer, Sam S. Karpiniec, Ah Y. Park, Chris van der Poel

**Affiliations:** ^1^ Department of Physiology, Anatomy & Microbiology School of Life Sciences La Trobe University Bundoora Victoria Australia; ^2^ Marinova Cambridge Australia

**Keywords:** endurance exercise, Fucoidan, muscle contraction, muscle fatigue, skeletal muscle

## Abstract

Fucoidan is a sulfated polysaccharide found in a range of brown algae species. Growing evidence supports the long‐term supplementation of fucoidan as an ergogenic aid to improve skeletal muscle performance. The aim of this study was to investigate the effect of fucoidan on the skeletal muscle of mice. Male BL/6 mice (*N* = 8–10) were administered a novel fucoidan blend (FUC, 400 mg/kg/day) or vehicle (CON) for 4 weeks. Treatment and control experimental groups were further separated into exercise (CON+EX, FUC+EX) or no‐exercise (CON, FUC) groups, where exercised groups performed 30 min of treadmill training three times per week. At the completion of the 4‐week treatment period, there was a significant increase in cross‐sectional area (CSA) of muscle fibers in fucoidan‐treated extensor digitorum longus (EDL) and soleus fibers, which was accompanied by a significant increase in tibialis anterior (TA) muscle force production in fucoidan‐treated groups. There were no significant changes in grip strength or treadmill time to fatigue, nor was there an effect of fucoidan or exercise on mass of TA, EDL, or soleus muscles. In gastrocnemius muscles, there was no change in mRNA expression of mitochondrial biogenesis markers PGC‐1α and Nrf‐2 in any experimental groups; however, there was a significant effect of fucoidan supplementation on myosin heavy chain (MHC)‐2x, but not MHC‐2a, mRNA expression. Overall, fucoidan increased muscle size and strength after 4 weeks of supplementation in both exercised and no‐exercised mice suggesting an important influence of fucoidan on skeletal muscle physiology.

## Graphical abstract

Fucoidan is a complex polysaccharide found in brown seaweed. While fucoidan has many bioactive properties, here we show that in mice, 4 weeks of supplementation with a novel blend of fucoidan species increased both size and strength of skeletal muscle.

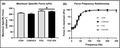

## 1 INTRODUCTION

Ergogenic aids are substances used by athletes to enhance athletic performance. Ergogenic aids are widely consumed by athletes and recreational adults with the belief that combining the consumption of supplements with exercise will promote gains in lean mass, improve recovery, decrease fatigue, and lead to overall improvements in physical performance (Erdman et al., 2007). Popular, affordable, and safe sports supplements including β‐alanine, creatine, and caffeine have been shown to improve pH buffering (Harris et al., 2006), increase PCr levels (Harris et al., 1992), and regulate extracellular K^+^ levels (Crowe et al., 2006). Identifying novel ergogenic aids to complement exercise will contribute to rapidly growing global supplements market.

Fucoidans are sulfated, complex, fucose‐rich polymers found in brown seaweeds and echinoderms (Fitton et al., 2015, 2019). Fucoidans have been shown to have multiple bioactive effects, including anti‐inflammatory (Lean et al., 2015), antitumor, and immune‐modulating properties (van Weelden et al., 2019). A recent study showed that supplementation with the same fucoidan blend as used in this study restores fecal lysozyme levels in high‐performance athletes (Cox et al., 2020). The influence of fucoidan on skeletal muscle physiology has remained relatively unexplored, although one recent study demonstrated that fucoidan, isolated from *Laminaria japonica*, improved the grip strength and swim endurance of male mice (Chen et al., 2014). However, the co‐ingestion of fucoidan with exercise has not been determined. With this in mind, we aimed to determine whether oral administration of a novel fucoidan blend could improve adaptation to moderate exercise and influence muscle function.

## 2 MATERIAL AND METHODS

### 2.1 Ethical approval

All procedures were approved by the Animal Ethics Committee of La Trobe University (AEC 18068). Animal care, maintenance, and procedures were conducted in accordance with the Australian Code of Practice for the Care and Use of Animals for Scientific Purposes. Twelve‐week‐old male C57BL/6 mice were purchased from the Walter and Eliza Institute of Medical Research (WEHI), and were housed in standard laboratory conditions (22 ± 2°C, relative humidity of 55 ± 8%, 12 h light/dark cycle) with access to water and a standard chow diet ad libitum.

### 2.2 Fucoidan preparation

A blended fucoidan extract derived from *Undaria pinnatifida* and *Fucus vesiculosus* was provided by Marinova Pty Ltd (Table 1). The proprietary aqueous extract was designed for ingestion, with a standardized fucoidan content >85% as determined by previously reported methods (Fitton, Stringer, Park, et al., 2019; Fitton, Dell'Acqua, et al., 2015). The carbohydrate profile was determined using a GC‐based method for the accurate determination of individual monosaccharide ratios in a sample. This method relies on the preparation of acetylated alditol derivatives of the hydrolyzed samples (Morvai‐Vitányi et al., 1993). The uronic acid content was determined by spectrophotometric analysis of the hydrolyzed compound in the presence of 3‐phenylphenol, based on a method described by (Filisetti‐Cozzi & Carpita, 1991). Sulfate content was analyzed spectrophotometrically using a BaSO_4_ precipitation method (BaCl_2_ in gelatin), based on the work of Dodgson (Dodgson & Price, 1962), and cations, including Na^+^, K^+^, Ca^2+^, and Mg^2+^, were determined by Flame Atomic Absorption Spectroscopy.

**TABLE 1:** Absolute mass percentages of components of fucoidan extract

Neutral carbohydrates (%)	Sulfate (%)	Counter‐ions (%)	Polyphenols (%)	Uronic acids (%)	Peak MW (kDa)
50.5	18.6	7.0	11.2	4.6	91.7 kDa

Fucose	Xylose	Galactose	Glucose	Arabinose	Rhamnose
28.36	3.68	9.83	7.45	0.70	0.47

This extract is polydisperse, containing molecular weight fractions ranging from 5 kDa to above 1000 kDa. Carbohydrate breakdown (mass %) of neutral carbohydrates

### 2.3 Treatment groups

Mice were randomly allocated to groups consisting of either vehicle control with no‐exercise (CON, *N* = 8), fucoidan with no‐exercise (FUC, *N* = 10), vehicle control plus exercise (CON+EX, *N* = 10), or fucoidan plus exercise (FUC+EX, *N* = 10). Fucoidan was administered daily via oral gavage at a dose of 400 mg/kg per day in injectable water (Lean et al., 2015), for a total of 4 weeks. Previous clinical research studies have used a daily dose of fucoidan of between 1 and 3 g per day (Cox et al., 2020; Irhimeh et al., 2007). To calculate the human equivalent dose (HED), we used the following formula from the US Food and Drug Administration: assuming a human weight of 60 kg, the HED for 2 (g)÷60 (kg) = 0.033 × 12.3 = a mouse dose of 400 mg/kg (Nair & Jacob, 2016). Mice allocated to the control groups received a daily dose of injectable water via oral gavage.

### 2.4 Fatigue test

Prior to all the treatment period, the mice were familiarized to running on a motorized rodent treadmill (model Exer 3/6, Columbus Instruments) set with zero incline. On the first day of familiarization, the mice were placed on the treadmill with the belt speed stationary for 10 min to explore the new environment. The following day, the mice were placed on the treadmill for another 10 min of exploration followed by the treadmill moving at a pace of 5 m/min for 10 min. On the third day, mice were placed on the treadmill for 5 min of stationary exploration followed by 10 m/min for 10 min. On the fifth day, mice underwent an exhaustion fatigue test which involved 5 min on the stationary treadmill, followed by 5 min at 10 m/min. After this 5‐min pace, the treadmill speed was increased by 2 m/min every 2 min until the mouse could no longer sustain the exercise as defined as the inability of the animal to run on the treadmill for 10 s, despite mechanical prodding. The exhaustion fatigue test was repeated at the completion of the 4‐week training protocol and was based on protocols outlined previously (Castro & Kuang, 2017). Running time (min) and distance covered (m) pre‐treatment were compared to time running and distance covered post‐treatment period.

### 2.5 Exercise protocol

Mice allocated to the moderate exercise group followed a protocol modified from previously published protocol for forced treadmill running in mice (Schmitt et al., 2018), and the maximal exercise capacity of 10‐ to 12‐week‐old male C57BL/6 mice (Ferreira et al., 2007). Our moderate exercise protocol consisted of mice running on a treadmill (model Exer 3/6, Columbus Instruments), three times a week (Monday, Wednesday, and Friday) for the 4‐week treatment period, at a speed of 15 m/min for 30 min. On each exercise day, mice ran at a speed of 15 m/min (60%–70% exercise capacity) for 30 min.

### 2.6 Grip strength test

In order to assess forelimb muscle strength grip, strength was assessed using the BIO‐GS3 Grip strength test apparatus (Bioseb In Vivo research Instruments) following Treat‐NMD protocols (Aartsma‐Rus & van Putten, 2014). Mice were encouraged to grip a metal bar attached to the apparatus and were then gently pulled backwards until they released. This was repeated five times with 5 min in between each recording, with the average of the five recordings being the grip strength recorded for each mouse. Absolute grip strength force (N) and normalized to body mas (N/g) were recorded both pre‐ and post‐treatment in all experimental groups.

### 2.7 In situ muscle function testing

At the end of the 4‐week treatment period, the isometric contractile properties of isolated fast‐twitch TA muscles were evaluated in situ, as previously described (Barker et al., 2017; Schertzer et al., 2006). Briefly, mice were anesthetized via intraperitoneal injection of sodium pentobarbitone (80 mg/kg). Once unresponsive to tactile stimuli, the distal portion of the TA muscle and its tendon were exposed. The distal tendon was tied with a top and bottom knot using two lengths of 5.0 braided surgical thread (Fine Science Tools), then severed, and the TA was dissected free from surrounding tissue. The sciatic nerve was exposed by parting the biceps femoris muscle above the knee joint. The mouse was secured proximally via a pin behind the patella tendon and a distal foot clamp on the heated platform (37°C) of an in situ Mouse Apparatus (809B, Aurora Scientific). The distal end of the TA was tied firmly to a lever arm attached to an isometric force transducer. Throughout the experiment, warmed physiological saline (0.9%) was applied to the exposed muscle and nerve tissue.

The TA was stimulated with supramaximal square wave pulses (10 V, 0.2 ms duration) to the sciatic nerve using a custom‐built hook electrode coupled to a stimulator (701C stimulator, Aurora Scientific). All stimulation parameters and contractile responses were controlled and measured using Dynamic Muscle Contraction 611A software (DMC, Aurora Scientific), with on‐board controller interfaced with the transducer control/feedback hardware. The contractile function testing protocol began with the determination of optimal muscle length (Lo) via micromanipulations of muscle length and a series of isometric twitch (Pt, 1 Hz) contractions. Maximum isometric tetanic force (Po) was determined from the force frequency curve (FFC)—1, 10, 20, 30, 40, 50, 80, 100, 150, and 200 Hz—with 2 min rest between each stimulation. Po values were normalized to muscle cross‐sectional area (CSA), determined from muscle fiber length and mass, and expressed as specific force (sPo, KN/m^2^) (Schertzer et al., 2006). Muscle fiber length (Lf) was determined by multiplying Lo by the previously determined Lf/Lo ratio of 0.6 for the TA (Schertzer et al., 2006). Following completion of the FFC, TA muscles were rested for 4 min. Then, to assess fatigability, muscles were stimulated at 100 Hz once every 5 s for 4 min. The extent of TA muscle fatigue was determined from the last contraction produced relative to the initial contraction.

Following completion of contractile function testing, mice were euthanized by cardiac puncture and the TA, fast‐twitch extensor digitorum longus (EDL), and slow‐twitch soleus muscles were excised, trimmed of tendons, weighed, embedded in optimal cutting temperature compound (Tissue‐Tek OCT Compound, ProSciTech), and frozen in thawing isopentane for histological analysis. The gastrocnemius muscle was excised for PCR analysis.

### 2.8 Histology for muscle morphology

Frozen gastrocnemius cross‐sections (8 µm thick) were cut from the mid‐belly region of EDL and soleus muscles and stained with hematoxylin and eosin (H&E) for quantitative assessment of muscle fiber size as per guidelines published by Treat‐NMD. Frozen sections were thawed and fixed for 3 min in 70% ethanol and stained with Mayer's H&E (Amber scientific MH‐2‐5L and EOS1‐1L, respectively) according to previously reported histological procedures (Liu et al., 2013). Representative digital images of H&E‐stained muscle were captured at 200× magnification (DM1000 upright microscope, Leica). Muscle fiber CSA was determined using ImageJ software (NIH). For each muscle, approximately 200 fibers were manually circled from six separate images. Muscle fiber CSA was then exported to Microsoft Excel to determine CSA per muscle type per experimental group.

### 2.9 Real‐time quantitative PCR (qPCR)

Frozen gastrocnemius muscle samples were homogenized individually in PureZol™ (Bio‐Rad Laboratories Inc.) using an IKA®Ultra‐Turrax® disperser (Sigma‐Aldrich) with 3 × 10 s bursts while on ice, and RNA was extracted using the spin protocol in Aurum™ Total RNA Fatty and Fibrous Tissue Kit (Bio‐Rad Laboratories Inc.) according to the manufacturer's instructions. RNA was transcribed into cDNA using the iScript™ cDNA synthesis kit (Bio‐Rad) according to the manufacturer's instructions. qRT‐PCR was performed using an iCycler Thermal Cycler (Bio‐Rad) with SsoFast™ EvaGreen® (Bio‐Rad). The expression level of all genes of interest (Table 2) was determined relative to the internal control gene, β‐2 microglobulin (β2M), and expressed as 2^−(deltaCT)^.

**TABLE 2:** Mouse primers for quantitative RT‐PCR

	Forward primer (5′–3′)	Reverse primer (5′–3′)
β2M	GTATGCTATCCAGAAAACCC	CTGAAGGACATATCTGACATC
*PGC‐1α*	GTATGTGAGATCACGTTCAAG	TTACCAACGTAAATCACACG
*Nrf‐2*	CTAGCCTTTTCTCCGCCTTT	GAGGCTACTTGCAGCAGAGG
*MHC‐2a*	AAGCGAAGAGTAAGGCTGTC	GTGATTGCTTGCAAAGGAAC
*MHC‐2x*	CACCGTCTGGATGAGGCTGA	TGTTTGCGCAGACCCTTGATAG
*Myogenin*	CTGCCACAAGCCAGACTC	GACTCCATCTTTCTCTCCTCA
*VEGF*	TAGAGTACATCTTCAAGCCG	TCTTTCTTTGGTCTGCATTC

### 2.10 Statistical analyses

All results are presented as mean ± SEM. The statistical analyses were performed to test for differences between vehicle control no‐exercise (CON), fucoidan no‐exercise (FUC), vehicle control + exercise (CON+EX), and fucoidan + exercise (FUC+EX). Experimental data were analyzed using a two‐way ANOVA, with the factors being exercise and fucoidan. When appropriate, Tukey post hoc analyses were performed. All statistical analyses were performed using GraphPad Prism v8, with *p* < .05 being considered statistically significant.

## 3 RESULTS

### 3.1 Fucoidan does not influence adaptation to exercise

**FIGURE 1:**
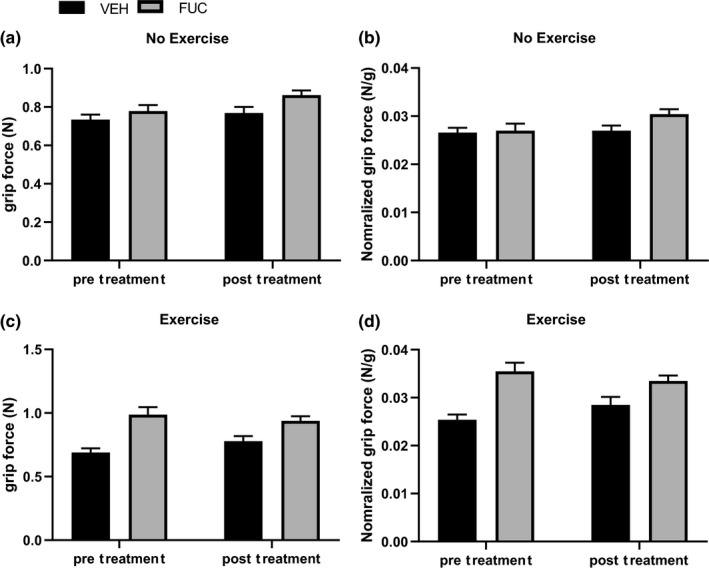
Effects of exercise and fucoidan on forelimb grip strength and endurance. Comparing pre‐treatment to post‐treatment period, there was no effect of vehicle (VEH) or fucoidan (FUC) treatment on absolute grip strength (a) or grip strength normalized to body mass (b) in either no‐exercise groups. Four weeks of moderate exercise did not significantly affect grip strength (c) or grip strength normalized to body mass (d) in either VEH+EX or FUC+EX groups. Data are expressed as means ± SEM, *n* = 8 for control, *n* = 10 for fucoidan, *n* = 10 for control + exercise, and *n* = 10 for fucoidan + exercise

Fucoidan has been hypothesized to stimulate appetite and cause an increase in body weight (Chen et al., 2014); however, in the present study, no significant effect on body weight was observed for the duration of the 4‐week treatment period between any of the groups (Table 3). The two physical performance measures to determine whether fucoidan improved adaptation to exercise were forelimb grip strength and a time to fatigue treadmill test. These tests were conducted before and after 4 weeks of daily fucoidan or vehicle control treatment. In the no‐exercise groups (Figure 1a,b), there was no change from pre‐ to post‐treatment period in the absolute grip strength (Figure 1a) or normalized to body weight (Figure 1b). In contrast to previous studies (Chen et al., 2014), there was no improvement in grip strength parameters in either the FUC or FUC+EX groups (Figure 1c,d). Consistent with previous studies (Kim et al., 2020), treadmill exercise did not significantly improve grip strength in the VEH experimental groups (Figure 1c,d).

**TABLE 3:** Mouse anthropometrics and contractile properties of TA muscle

	Control	Control + exercise	Fucoidan	Fucoidan + exercise
Body mass BM (g)	29.51 ± 0.56	28.29 ± 0.46	29.31 ± 0.75	28.14 ± 0.59
Body mass gain (%)	6.71 ± 1.98	4.45 ± 3.37	1.52 ± 4.48	1.72 ± 2.46
TA mass MM (mg)	51.81 ± 1.37	53.94 ± 2.62	56.15 ± 1.45	55.10 ± 2.17
MM:BW ratio	1.76 ± 0.06	1.91 ± 0.10	1.92 ± 0.10	1.96 ± 0.08
P_t_ (mN/mm^2^)	57.3 ± 2.05	61.82 ± 3.94	56.8 ± 3.87	58.46 ± 4.16
TPT (ms)	253.36 ± 10.35	246.77 ± 5.64	245.8 ± 7.60	251.16 ± 8.44
Dx/Dt (mN/ms)	32.14 ± 1.11	30.11 ± 0.99	32.45 ± 1.04	29.59 ± 2.36
½ RT (ms)	18.30 ± 1.40	19.67 ± 1.8	17.02 ± 0.96	18.57 ± 2.13
Po (mN)	1610 ± 47.15	1595 ± 52.79	1728 ± 38.91	1683.16 ± 29.49
TA fatigue index	0.82 ± 0.01	0.81 ± 0.03	0.84 ± 0.03	0.82 ± 0.05
FS50 (Hz)	34.84 ± 1.44	34.93 ± 0.76	38.12 ± 0.75	38.99 ± 1.56
EDL CSA (µm^2^)	2853.11 ± 743.21	3047.69 ± 643.68	3400.06 ± 868.85*	3272.43 ± 758.09*
Soleus CSA (µm^2^)	1810.95 ± 636.64	1730.20 ± 695.57	2034.19 ± 766.55*	3272.48 ± 758.09*

Note

Values shown are mean ± SEM. *n* = 8 for control, *n* = 10 for fucoidan, *n* = 10 for control + exercise, and *n* = 10 for fucoidan + exercise.

Abbreviations: TA, tibialis anterior, CSA, cross‐sectional area, P_t_, maximum twitch force, TPT, time to peak twitch, Dx/Dt, rate of twitch force development, ½ RT, one‐half relaxation time, P_o_, maximum tetanic force, FS50, stimulation frequency to achieve 50% of maximal force.

* *p* < .05 compared to control.

**FIGURE 2:**
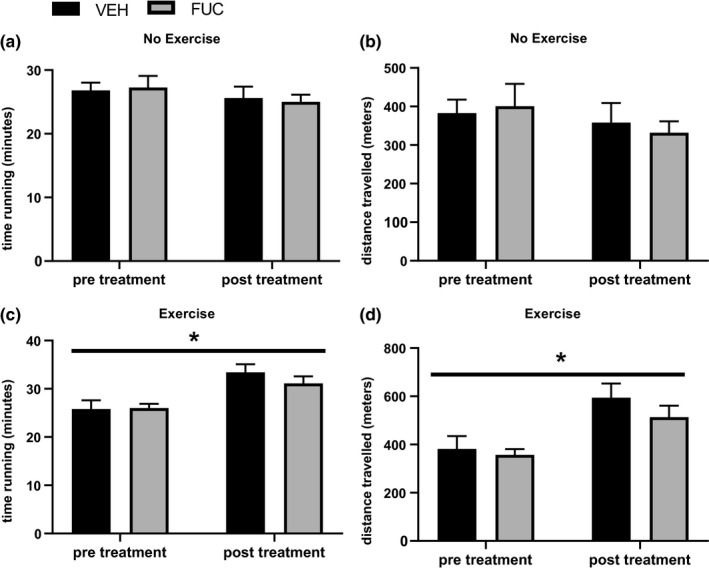
Effects of exercise and fucoidan on treadmill running endurance. In the VEH and FUC groups that did not exercise, there was no change in time running on the treadmill (a) or distance covered (b) pre‐ or post‐4‐week treatment period. Following 4 weeks of moderate exercise, VEH+EX and FUC+EX had significantly improved both time running (c) and distance traveled (d). Data are expressed as means ± SEM, *p < .05 – main effect for exercise; two‐way ANOVA. *n* = 8 for control, *n* = 10 for fucoidan, *n* = 10 for control + exercise, and *n* = 10 for fucoidan + exercise

For the VEH and FUC groups that did not exercise, there were no improvements in treadmill running time (Figure 2a) or distance traveled (Figure 2b). Importantly, when comparing the treadmill fatigue test between the pre‐ and post‐treatment period, 3 × 30‐min treadmill exercise bouts for 4 weeks significantly increased both time on treadmill (Figure 2c) and distance run (Figure 2d) (*p* < .05, main exercise effect, two‐way ANOVA). These improvements were seen in both VEH+EX and FUC+EX groups and treatment with fucoidan did not increase or decrease the positive training effect (Figure 2c,d).

### 3.2 Fucoidan improves specific force production of tibialis anterior muscles in situ

**FIGURE 3:**
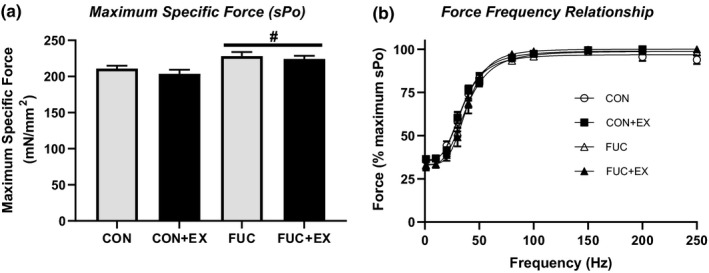
Effects of exercise and fucoidan on tibialis anterior (TA) strength. (a) Exercise had no effect on muscle strength normalized to muscle size (sPo); however, both experimental groups receiving fucoidan demonstrated significantly stronger sPo. (b) There was no effect of exercise or fucoidan on force frequency relationship of TA muscle. ^#^ *p* < .05 – main effect for fucoidan, two‐way ANOVA. *n* = 8 for control, *n* = 10 for fucoidan, *n* = 10 for control + exercise, and *n* = 10 for fucoidan + exercise

When TA contractile function was assessed with an intact blood supply in situ, neither fucoidan or exercise had a significant effect on absolute TA force (Po), twitch force (Pt), time to peak tension (TPT), rate of force production (+Dx/Dt), or half relaxation time (½RT) (Table 3). However, when tetanic force was normalized to muscle mass and expressed as specific force (sPo), supplementation with fucoidan did show a main effect for fucoidan treatment on sPo (Figure 3a, *p* < .05 main effect for fucoidan, two‐way ANOVA) with a Tukey post hoc test showing that sPo was significantly increased in FUC and the FUC+EX experimental groups (Figure 3a, *p* < .05). There was no significant difference between sPo in CON or CON+EX experimental groups. To determine whether exercise or fucoidan affected in situ force output in response to changes in stimulation frequency, force was expressed as a percentage of maximal Po at 200 Hz (Figure 3b). Neither exercise nor the supplementation with fucoidan had a significant effect on the relative submaximal force production, as measured by the frequency that produced 50% maximum force (FS50), in TA muscle (Table 3).

There is a tight association between muscle fiber CSA and force production (Narici et al., 1989). Interestingly in this study, there was a significant increase in CSA of fast‐twitch EDL and slow‐twitch soleus fibers (Table 3) in both the FUC and FUC+EX experimental groups (*p* < .05, two‐way ANOVA), while there was no significant change in fiber CSA for either the CON or CON+EX experimental groups over the 4 weeks of treatment.

### 3.3 Fucoidan does not influence transcription factors associated with mitochondrial biogenesis

**FIGURE 4:**
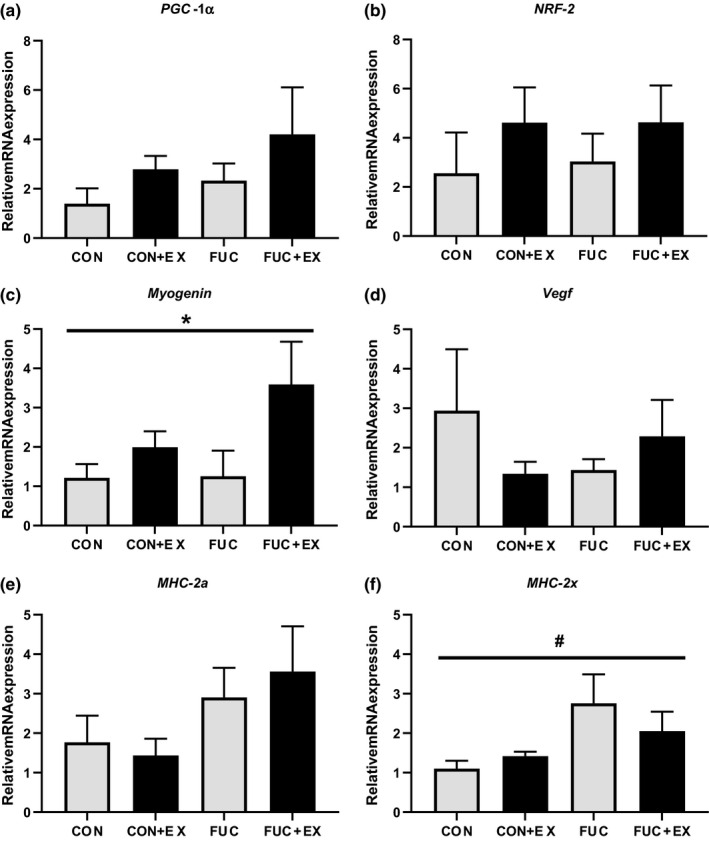
Effects of exercise and fucoidan on mRNA expression in gastrocnemius. Markers of mitochondrial biogenesis (a,b), angiogenesis (c), myogenesis (d), and myosin heavy chain isoform (e,f). *p < .05 – main effect for exercise, ^#^ *p* < .05 – main effect for fucoidan; two‐way ANOVA. *n* = 8 for control, *n* = 10 for fucoidan, *n* = 10 for control + exercise, and *n* = 10 for fucoidan + exercise

The transcriptional coactivators PGC‐1α and Nrf‐2 are key transcription factors in mediating exercise training‐induced adaptations such as increased mitochondrial biogenesis and increased mitochondrial function (Merry & Ristow, 2016a, 2016b). The present study found that 4 weeks of treadmill exercise did not significantly increase mRNA levels of either PGC‐1α (Figure 4a) or Nrf‐2 (Figure 4b). Likewise, mRNA levels of PGC‐1α and Nrf‐2 were not significantly affected by 4 weeks of fucoidan treatment.

Previous studies have shown evidence that modulating expression of the transcription factor myogenin can induce changes in muscle phenotype as well as increasing oxidative metabolism of skeletal muscle (Flynn et al., 2010; Hughes et al., 1993). Here we show that there was no significant effect of fucoidan on myogenin expression (Figure 4c); however, we did observe a main effect of exercise on myogenin expression (*p* < .05, main effect for exercise, two‐way ANOVA).

VEGF is responsible for maintenance and increasing capillary density of skeletal muscle and is increased after exercise training (Gustafsson et al., 2002). In the present study, we found no evidence that either exercise or fucoidan influenced VEGF expression (Figure 4d).

To determine whether the increase in force production (Figure 3a) and increased CSA (Table 3) observed in response to fucoidan were associated with a shift in fiber type, gene expression of myosin heavy chain (MHC) isoforms within muscle fibers, specifically the fast‐twitch isoforms MHC2a (Figure 4e) and MHC2x (Figure 4f), were measured. In this study, there was no significant effect of either exercise or fucoidan on MHC2a expression (Figure 4e). There was a main effect on the expression of MHC2x associated with fucoidan treatment (*p* < .05, main effect fucoidan, two‐way ANOVA), but not exercise (Figure 4f). Despite a main effect for fucoidan (*p* < .05, two‐way ANOVA), there was no significant difference in MHC2x expression levels observed between groups.

## 4 DISCUSSION

Identification of an ergogenic aid that can increase the beneficial effects of exercise would appeal to athletes of all types. This study demonstrates that in contrast to previous studies, oral administration of fucoidan did not upregulate gene levels of transcription factors associated with mitochondrial biogenesis and did not improve adaptation to endurance training. Despite no improvements in measures of endurance, daily oral administration of fucoidan significantly increased the force‐producing properties of the TA muscle in situ. This increased force production in the TA was accompanied by an increase in fiber CSA in both fast‐twitch EDL and slow‐twitch soleus fibers and a significant treatment effect of fucoidan on mRNA expression of MHC2x in gastrocnemius muscles.

Previous work showed that supplementation with a single fucoidan species isolated from *Laminaria japonica* and orally administered to mice for 3 weeks increased grip strength and swimming endurance in a dose‐dependent manner (Chen et al., 2014). These improvements were associated with changes in blood lactate and blood glucose levels. Importantly, these positive effects of fucoidan on muscle performance were established in the absence of any training. In our study, we used a novel blend of fucoidan that did not replicate the effects of the single fucoidan species shown to positively affect muscle performance previously (Chen et al., 2014). It is known that the yield of fucoidan varies depending on the species, season, geographical origin, and extraction method (Fitton, Stringer, et al., 2015), suggesting that species of brown algae used is an important consideration when comparing outcomes. Using the same extraction methods, the comparative compositions of the species used previously (Chen et al., 2014), *Laminaria japonica*, and the two species used here are described (Fitton et al., 2019). We can hypothesize that fucoidan extracted from *Laminaria japonica* influences pathways associated with muscle lactate and glucose metabolism, whereas those used in this study, *Undaria pinnatifida* and *Fucus vesiculosus*, seem to influence pathways associated with muscle hypertrophy.

An important finding in this study is that supplementation of fucoidan during a moderate exercise protocol did not amplify adaptation to exercise. We acknowledge that the exercise protocol utilized in this study should be considered mild; however, we did clearly see adaptation to exercise with both exercise groups improving both run time and distance covered during a treadmill fatigue test. As our fucoidan blend did not improve adaptation to moderate exercise, it seems that our blend of fucoidan does not influence exercise‐induced adaptation pathways that include fluxes in intracellular Ca^2+^, changes to energy metabolism, or oxidative stress. However, we acknowledge that the exercise protocol utilized in this study was a moderate exercise protocol and as such it would be important for future studies to incorporate a more intense exercise protocol to sufficiently determine whether fucoidan can enhance the benefits of exercise. Another potential limitation of this study is the investigation of pathways in different muscle groups. Muscle fibers from different muscle groups contain different MHC isoform profiles, and although the current study found no evidence for isoform‐specific effects of fucoidan on muscle fibers, future studies would benefit from investigating biochemical, functional, and histological changes in the same muscle groups.

The findings of this study build on existing evidence suggest that fucoidan has positive effects on skeletal muscle function and should be further investigated for its use as a dietary supplement for exercise and athletic performance. It is also interesting to consider fucoidan as a supplement that has potential positive effects in musculoskeletal disorders that have decreased metabolic activity or increase muscle atrophy. The positive effects of consistent physical activity on pathologies, such as sarcopenia, diabetes and obesity, and cardiovascular disease, are undeniable. However, physical exercise is not always attainable and as such supplements that mimic or potentiate the effect of exercise are important.

In type 2 diabetes, a decrease in muscle mass decreases the metabolic capacity of body which exacerbates the T2DM pathology (Andreassen et al., 2006). Previous studies have already demonstrated that fucoidan can increase insulin‐stimulated glucose uptake (Sim et al., 2019) and prevent β‐cell dysfunction (Yu et al., 2017). It would be interesting to further investigate the association between fiber type and insulin‐stimulated glucose uptake as type 2 muscle fibers show greater insulin‐stimulated glucose uptake compared to other fiber types (Mackrell et al., 2012).

Another muscle wasting pathology that has received significant attention is cancer cachexia. Cancer cachexia is the progressive loss of muscle mass and is thought to contribute to patient's response to chemotherapy contributing to 20% of all cancer deaths (Tisdale, 2005). In a recent study, it was demonstrated that the addition of the marine brown alga, *Sargassum hemiphyllum*, to a cocktail of chemotherapy drugs reduced gastrocnemius and soleus atrophy and improved survival rate by 20% in a mouse model of bladder cancer (Chen et al., 2016). The reduction in cachectic symptoms was attributed to fucoidan reducing the negative regulator of muscle growth, myostatin, as well as key mediators of protein degradation, atrogin‐1 and Murf‐1. The results of the present study appear to support the ability of fucoidan to increase muscle mass, not solely by influencing the tumor, but also by influencing skeletal muscle fiber type and promoting a shift to larger CSA muscle fibers.

## 5 PERSPECTIVES AND CONCLUSIONS

In this study, we have demonstrated that dietary supplementation with fucoidan does not potentiate the effects of a moderate exercise training protocol in mice. However, the results are clear that fucoidan improved muscle strength of fast‐twitch muscles and increased fiber CSA for both fast‐ and slow‐twitch muscle types. Our findings highlight the potential of fucoidan in the field of sports supplementation as well as musculoskeletal pathologies that could improve overall muscle health and performance.

## CONFLICT OF INTEREST

The current study was sponsored in part by Marinova Pty Ltd. J. Helen Fitton, Damien Stringer, Sam Karpiniec, and Ahyoung Park are employees of Marinova Pty Ltd. The remaining authors have no conflict to declare.

## AUTHOR CONTRIBUTIONS

C.v.d.P., J.E.C., and C.J.T. conceived and designed research; S.E.M, B.K.T., and C.v.d.P. performed experiments; S.E.M., C.v.d.P., B.K.T., and C.J.T analyzed data; C.v.d.P and J.E.C. interpreted the results of the experiments; S.E.M., B.K.T., and C.v.d.P. prepped figures; J.E.C., J.H.F, A.Y.P, and C.v.d.P. drafted the manuscript; B.K.T., J.E.C., C.J.T., J.H.F, D.N.S., A.Y.P., and C.v.d.P. edited and revised the manuscript; S.E.M., J.E.C., C.J.T., B.K.T., J.H.F, A.Y.P., D.N.P., S.S.K., and C.v.d.P approved the final version of the manuscript.
